# Methylene blue decreases mitochondrial lysine acetylation in the diabetic heart

**DOI:** 10.1007/s11010-017-2993-1

**Published:** 2017-03-16

**Authors:** Jessica M. Berthiaume, Chia-heng Hsiung, Alison B. Austin, Sean P. McBrayer, Mikayla M. Depuydt, Margaret P. Chandler, Masaru Miyagi, Mariana G. Rosca

**Affiliations:** 1 Department of Physiology & Biophysics, Case Western Reserve University, Cleveland, OH USA; 2 Department of Foundational Sciences, Central Michigan University College of Medicine, 2630 Denison Drive, Research Building Room 105, Mount Pleasant, MI 48858 USA; 3 Center for Proteomics and Bioinformatics, Case Western Reserve University, Cleveland, OH USA

**Keywords:** Cardiomyopathy, Diabetes, Mitochondria, Methylene blue, Fatty acid oxidation, Lysine acetylation

## Abstract

Diabetic cardiomyopathy is preceded by mitochondrial alterations, and progresses to heart failure. We studied whether treatment with methylene blue (MB), a compound that was reported to serve as an alternate electron carrier within the mitochondrial electron transport chain (ETC), improves mitochondrial metabolism and cardiac function in type 1 diabetes. MB was administered at 10 mg/kg/day to control and diabetic rats. Both echocardiography and hemodynamic studies were performed to assess cardiac function. Mitochondrial studies comprised the measurement of oxidative phosphorylation and specific activities of fatty acid oxidation enzymes. Proteomic studies were employed to compare the level of lysine acetylation on cardiac mitochondrial proteins between the experimental groups. We found that MB facilitates NADH oxidation, increases NAD^+^, and the activity of deacetylase Sirtuin 3, and reduces protein lysine acetylation in diabetic cardiac mitochondria. We identified that lysine acetylation on 83 sites in 34 proteins is lower in the MB-treated diabetic group compared to the same sites in the untreated diabetic group. These changes occur across critical mitochondrial metabolic pathways including fatty acid transport and oxidation, amino acid metabolism, tricarboxylic acid cycle, ETC, transport, and regulatory proteins. While the MB treatment has no effect on the activities of acyl-CoA dehydrogenases, it decreases 3-hydroxyacyl-CoA dehydrogenase activity and long-chain fatty acid oxidation, and improves cardiac function. Providing an alternative route for mitochondrial electron transport is a novel therapeutic approach to decrease lysine acetylation, alleviate cardiac metabolic inflexibility, and improve cardiac function in diabetes.

**Electronic supplementary material:**

The online version of this article (doi:10.1007/s11010-017-2993-1) contains supplementary material, which is available to authorized users.

## Introduction

Diabetic cardiomyopathy evolves from hypertrophy and fibrosis with myocardial stiffness to drive cardiac dysfunction and heart failure in humans [1, 2]. This progression has been recapitulated in a number of animal models [3, 4]. Even in well-controlled diabetic patients, signs of cardiomyopathy are often present [1, 2, 5]; however, targeted therapies do not exist.

The molecular mechanisms regulating diabetic cardiomyopathy are unknown. Cardiac metabolic inflexibility with excessive reliance on mitochondrial fatty acid (FA) oxidation for ATP production is considered a key pathogenic mechanism [3]. This metabolic inflexibility is due to either insulin lack/insensitivity that decreases glucose uptake and oxidation or metabolic remodeling that enhances mitochondrial FA oxidation. Interestingly, overreliance on lipids precedes cardiac insulin resistance in obese human subjects [6] and rodents [7], and is an early event following increased FA availability [8], suggesting that the metabolic rigidity of the diabetic heart is linked to increased FA availability rather than decreased cardiac insulin activity.

Myocardial overreliance on FA oxidation is preceded by an increase in circulating FA in diabetes (DB) [9]. While cardiac FA uptake increases in DB [10], how mitochondrial FA oxidation is up-regulated is unknown. Post-translational modification by the metabolic end-product, acetyl-CoA, can regulate cardiac metabolic enzymes in a dynamic, reversible manner. Increased lysine acetylation of mitochondrial proteins and insulin signaling machinery [11] has been suggested to drive early changes in substrate selection. Whereas lysine acetylation depends on acetyl-CoA, deacetylation is mediated by sirtuins and deacetylases are regulated by NAD^+^. Lysine acetylation-mediated metabolic regulation has been the aim of numerous studies, though whether protein lysine acetylation plays a role in the etiology of metabolic inflexibility in the diabetic heart remains unclear. Individual FA oxidation enzymes are sirt3 substrates, and sirt3 knockout leads to their inhibition [12]. In contrast, high fat feeding [11] and type 1 diabetes [13] lead to hyper-acetylation of FA β-oxidation enzymes, and increases cardiac lipid oxidation. Although metabolic inflexibility in the diabetic heart is a recognized phenomenon, how the overreliance on FA oxidation disrupts cardiac physiology is unknown. In murine models of type 2 diabetes [14, 15] and obese humans [16] increased mitochondrial FA oxidation diminishes cardiac mechanical efficiency [17] by FA-induced uncoupling of oxidative phosphorylation whereas in models of type 1 diabetes, mitochondrial uncoupling is not evident [18].

Substrate oxidation generates reducing equivalents for use by the mitochondrial electron transport chain (ETC) to drive ATP synthesis. Alternative electron transport pathways were reported to rescue mitochondrial dysfunction induced by ETC deficiencies [19]. Chemical moieties capable of electron shuttling include the aromatic compound methylene blue (MB), a FDA-approved drug used for almost a century to treat a wide spectrum of ailments [20]. Its ability to participate in redox reactions makes it an attractive potential therapeutic drug to ameliorate the consequences of ETC defects. MB accepts electrons from complex I [21, 22], whereas reduced MB (MBH_2_) donates electrons to downstream ETC subunits, such as cytochrome c [19, 23]. In light of MB’s ability to participate in redox reactions we hypothesized that treatment with MB would decrease the NADH/NAD^+^ ratio and facilitate lysine deacetylation in the diabetic heart.

## Materials and methods

### Reagents

Unless otherwise specified, all reagents were of the highest purity grade and purchased from Sigma-Aldrich.

### Animal models

All animal experiments were conducted in accordance with the Guide for the Care and Use of Laboratory Animals published by the US National Institutes of Health (NIH Publication No. 85 − 23, revised 2011), and approved by the Case Western Reserve University and Central Michigan University Institutional Animal Care and Use Committees. Adult, 2-month-old, male Lewis rats were randomly assigned to an experimental group: control (CTL), control+MB (CTL+MB), DB, or DB+MB. DB was induced by intraperitoneal injection of streptozotocin (55 mg/kg body wt); DB was confirmed by the onset of hyperglycemia assessed in the tail vein blood (~2 weeks after streptozotocin administration). Low doses (0–0.2) of insulin (neutral protamine hagedorn) was given subcutaneously up to 3× per week in order to avoid a severe hypercatabolic state and ketosis without preventing hyperglycemia and glucosuria. MB was administered in drinking water immediately following streptozotocin injection, and water intake was monitored weekly. MB shows opposite effects at low and high doses (hormetic effect) [24]. Low MB concentrations favor reduction, whereas at higher concentrations MB may “re-route” electrons away from the ETC, disrupting the redox balance and acting as a pro-oxidant [25]. Our dose selection was based on the reported dose of 1–10 mg/kg in acute intraperitoneal administration that improved memory in mice [26–28]. In addition, the oral dose of 10 mg/kg leads to an optimal concentration in the heart tissue [29].

DB animals developed polydipsia, thus MB oral dosing was adjusted to standardize treatment based on water intake and individual rat weight to deliver 10 mg MB/kg/day in CTL and DB rats. Over the 11 weeks water intake was (ml/day); CTL = 25.1 ± 0.6 to 23.9 ± 2.0, CTL+MB 21.25 ± 1.5 to 19.2 ± 1.5, DB 129.2 ± 1.7 to 14 to 1.1 ± 2.1, and DB+MB 130 ± 2.2 to 134.9 ± 1.5. MB treatment did not alter water intake of DB rats.

Cardiac function was assessed by two-dimensional guided M-mode, two-dimensional Doppler flow echocardiography, as described [13, 30, 31]. Hemodynamic parameters were further evaluated using a Millar pressure transducer catheter introduced into the LV via the right carotid artery [13, 31]. Following 11 weeks, rats were euthanized with pentobarbital (100 mg/kg); blood and the left ventricle (LV) were sampled for further analysis. Hyperglycemia was quantified by measuring blood glucose concentration (glucose dehydrogenase-based strips) and glycated hemoglobin (Variant II total GHb program; BioRad).

In a subset of experiments to investigate the role of sirtuin 3 (sirt3) in the deacetylating effect of MB, cardiac tissue was harvested from male, 6-month-old, sirt3 knockout (KO) mice (Jackson Laboratory) to isolate cardiac mitochondria for analysis in respiration studies.

### Immunoblotting

Denatured protein samples from LV tissue or cardiac mitochondria were separated by SDS–PAGE (4–12% Tricine gel, 50 µg/well), electroblotted onto PVDF membranes, and probed with primary (dilution 1:3000) and HRP-conjugated secondary antibodies (dilution 1:10,000). Membranes were developed using Amersham ECL™ substrates. Densitometric analysis was performed with Image J coupled with Multi Gauge V3.0 (Fujifilm Life Science) and the Licor Odyssey Fc imaging system (Biotech). Antibodies used were against acetyllysine, sirtuin 3, and glyceraldehyde 3-phosphate dehydrogenase (cell signaling), cytochrome c (mitosciences), and atrial natriuretic peptide (thermo fisher).

### Mitochondrial respiratory studies

Cardiac mitochondria were isolated from freshly harvested rat LV using a previously published protocol [32]. For experiments with sirt3 KO mice, a combined population of cardiac mitochondria was isolated from the whole mouse heart to maximize yield for subsequent analysis. Protein concentration was determined by the Lowry method. Mitochondria were investigated for respiratory properties as described [13, 33].

### NADH and NAD quantitation

Freshly isolated cardiac mitochondria were incubated as outlined for oxidative phosphorylation measurements. One milligram of cardiac mitochondrial protein was incubated for 5 min in either oxidative buffer alone (baseline) or supplemented with the respiratory substrate palmitoylcarnitine+malate (PC). Methylene blue was added during PC oxidation at the indicated concentrations. NAD and NADH were extracted and quantified using a kit according to the manufacturer’s instructions (cell technology).

### Sirtuin 3 activity

Sirt3 activity of frozen-thawed mitochondria isolated from control (untreated) rat hearts was assessed as deacetylation of a substrate peptide that emits fluorescence with an excitation emission of 340–440 nm using a kit according to manufacturer’s instructions (Sirt3 activity kit, Abcam).

### Mitochondrial fatty acid oxidation enzyme activities

The activities of medium- and long-chain acyl-CoA dehydrogenases (MCAD, LCAD) in cardiac mitochondria were measured using medium- and long-chain acyl-CoA substrates (octanoyl-CoA and palmitoyl-CoA), phenazine ethosulfate (intermediate electron acceptor from FADH_2_), and oxidized cytochrome c (final electron acceptor). Mitochondrial matrix was separated by centrifugation of freeze-thawed, sonicated, and Triton X-100-treated (detergent:protein 1:1) cardiac mitochondria. Hydroxyacyl-CoA dehydrogenase (HADH) was measured as the reverse reaction of 3-ketoacyl-CoA conversion to 3-hydroxyacyl-CoA with NADH as the electron donor. NADH oxidation was monitored as the decrease in absorption at 340 nm.

### Affinity purification of lysine-acetylated peptides for proteomic studies

To evaluate the impact of MB treatment on DB-induced acetylation, we used a proteomic approach according to a previously published protocol [13]. Briefly, 135 µg mitochondrial protein from three rats from each group was pooled, electrophoresed, and following separation the entire lane was subjected to in-gel trypsin digestion [34]. Tryptic peptides from DB and DB+MB samples were labeled with ^16^O and ^18^O [35], respectively, then mixed for differential isotopic analysis. Lysine-acetylated peptides were enriched by immunoprecipitation with anti-Ac-Lys antibody beads (ImmuneChem Pharmaceuticals), and analyzed by LC-MS/MS using an UltiMate 3000 LC system (Dionex Inc., Sunnyvale, California) interfaced with a Velos Pro Ion Trap/Orbitrap Elite hybrid mass spectrometer (Thermo Scientific, Bremen, Germany) [36]. Lysine-acetylated peptides were identified by comparing resultant MS/MS spectra against the Swiss-Prot database using Mascot database search software (version 2.4, Matrix Science, London, UK). Carbamidomethylation of cysteine was set as a fixed modification, whereas variable modifications included acetylation of ε-amino group of Lys, oxidation of methionine to methionine sulfoxide, and ^18^O labeling of C-terminal carboxyl group. The mass tolerance was set at 10 ppm for precursor ions and 0.8 Da for product ions. Strict trypsin specificity was applied with one missed cleavage allowed. The ^16^O/^18^O ratio of each peptide was calculated using Proteomics Tools software (available at https://github.com/shengqh/RCPA.Tools) [37].

### Statistical analysis

Data were evaluated for statistical differences among groups using student’s 2-tailed *t* test and a two-way ANOVA followed by Bonferroni’s multiple comparison test. Data are reported as mean ± SEM with data representing *n* = 3–7 per group. Significance was established at *p* < 0.05.

## Results

### Clinical, biochemical, echocardiographic, and hemodynamic characteristics

To best recapitulate the clinical setting of DB, we used a model that included management of DB rats with insulin to reduce hypercatabolism and avoid ketosis. Body weights of diabetic (DB) rats were lower than treatment-matched controls (CTL); however, DB group did not lose weight over the experimental period indicating that insulin prevented a hypercatabolic state (Table 1). Methylene blue (MB) treatment did not affect body weight in control (CTL+MB) or diabetic rats (DB+MB). DB rats exhibited hyperglycemia over the 11-week study as evidenced by increased fasting and non-fasting blood glucose concentrations FBG and NFBG, respectively (Table 1). MB treatment did not significantly affect NFBG or FBG, though a trend of an increase in NFBG was noted in DB+MB. HbA1c was also increased in the DB group as compared to treatment-matched control (DB versus CTL, ~38 mmol/mol vs. <9 mmol/mol, respectively) confirming the development of diabetes, and was not influenced by the MB treatment.

**Table 1:** Summary of clinical, biochemical, echocardiographic, and hemodynamic characteristics of control (CTL), control+methylene blue (CTL+MB), 11-week diabetic (DB), and 11-week diabetic+MB (DB+MB) rats

	CTL	CTL+MB	DB	DB+MB
Animal data and circulating metabolites				
BW_start_ (g)	285.0 ± 6.9	282.0 ± 9.9	285.8 ± 4.4	285.8 ± 3.4
BW_end_ (g)	359.0 ± 15.9	369.0 ± 16.5	273.0 ± 3.8*	265.0 ± 11.5*
NFBG (mg/dL)	98.6 ± 1.3	97.3 ± 4.5	400.2 ± 14.9*	456.8 ± 23.9*
FBG (mg/dL)	99.0 ± 0.5	95.3 ± 2.3	310.6 ± 10.8*	320.1 ± 8.9*
Hb A1C (%)	2.5 ± 0.1	2.5 ± 0.1	5.6 ± 0.1*	5.6 ± 0.2*
Echocardiographic data				
HR (bpm)	363 ± 10	354 ± 7	284 ± 11*	273 ± 14*
End-diastolic dia. (cm)	0.79 ± 0.02	0.77 ± 0.02	0.84 ± 0.02*	0.81 ± 0.01
End-systolic dia. (cm)	0.45 ± 0.03	0.45 ± 0.01	0.50 ± 0.03*	0.44 ± 0.01
Ejection time (msec)	73 ± 2	74 ± 2	105 ± 7*	108 ± 5*
Fraction shortening	0.44 ± 0.03	0.43 ± 0.01	0.41 ± 0.01	0.45 ± 0.01
End-diastolic vol. (ml)	0.52 ± 0.03	0.50 ± 0.03	0.64 ± 0.05*	0.57 ± 0.03
End-systolic vol. (ml)	0.09 ± 0.01	0.08 ± 0.01	0.13 ± 0.02*	0.10 ± 0.01
LV hemodynamics				
End-systolic vol. (μL)	132.8 ± 11.4	133.4 ± 14.7	199.5 ± 28.5*	149.6 ± 11.4^#^
End-diastolic vol. (μL)	477.7 ± 6.3	450.8 ± 27.2	579.7 ± 43.3*	486.8 ± 26.1^#^
Stroke vol. (µL)	380.1 ± 22.5	367.5 ± 18.3	454.9 ± 36.9*	419.0 ± 17.1
End-systolic pressure (mmHg)	118.9 ± 4.8	124.1 ± 6.4	98.6 ± 4.5*	109.0 ± 3.3*
End-diastolic pressure (mmHg)	6.3 ± 0.5	7.6 ± 0.9	9.3 ± 0.9*	8.8 ± 0.2*
Ejection fraction (%)	75.6 ± 1.6	78.0 ± 0.9	72.9 ± 2.7*	76.3 ± 1.3^#^
+dP/d*t* (mmHg/sec)	6829 ± 638	7580 ± 559	4464 ± 168*	4870 ± 401*
−dP/d*t* (mmHg/sec)	10,292 ± 1027	10,551 ± 1346	4734 ± 420*	4773 ± 407*
Power_max_ (mWatts/μL^2^)	5.4 ± 0.8	7.3 ± 1.1	2.85 ± 0.3*	4.2 ± 0.6^#^
Tau_weiss_ (msec)	10.1 ± 0.5	9.7 ± 0.4	19.2 ± 1.1*	19.0 ± 1.1*

*NFBG* non-fasting blood glucose, *FBG* fasting blood glucose, *MPI* myocardial performance index, *dV* diastolic volume, *sV* systolic volume.

*p < 0.05 CTL (*N* = 7) versus DB (*N* = 7), and ^#^ *p* < 0.05 DB (*N* = 7) versus DB+MB (*N* = 7). Mean ± SEM. Full list of measured parameters can be found in Additional file: Table S1

Cardiac function assessed by echocardiography revealed that heart rate was decreased in the DB groups as compared to the respective CTL leading to prolonged ejection and contraction/relaxation times which were not altered by MB treatment. Fractional shortening was not altered across the groups despite a modest trend towards a decrease in the DB group. LV end-systolic and -diastolic dimensions were increased in DB indicating cardiac remodeling. Ventricular dimensions in the DB+MB group were not significantly different from CTL+MB as measured by echocardiography. In contrast, atrial natriuretic peptide (ANP), a marker of pathological hypertrophy, was slightly increased in the diabetic heart and normalized by the MB treatment (Additional file: Figure S1). In vivo LV hemodynamic analyses showed that end-systolic and -diastolic pressures along with load-dependent indices of contractility, +/−dP/d*t*, were diminished in the hearts of DB rats regardless of MB treatment (Table 1). However, most if not all hemodynamic parameters trended towards improvement in DB+MB as compared to the DB group (for all cardiac functional parameters measured, see Additional file: Table S1). Additionally, end-systolic and -diastolic volumes and stroke volumes were altered in DB rats, and were significantly improved in the DB+MB group consistent with our echocardiographic data. Furthermore, there was a diabetes-induced decrease in ejection fraction that was significantly improved in DB+MB. Together these data indicate that chronic MB treatment leads to improved cardiac function in this model of diabetic cardiomyopathy.

### MB decreases protein lysine acetylation by increasing mitochondrial NAD^+^ and sirt3 activity

**Fig. 1:**
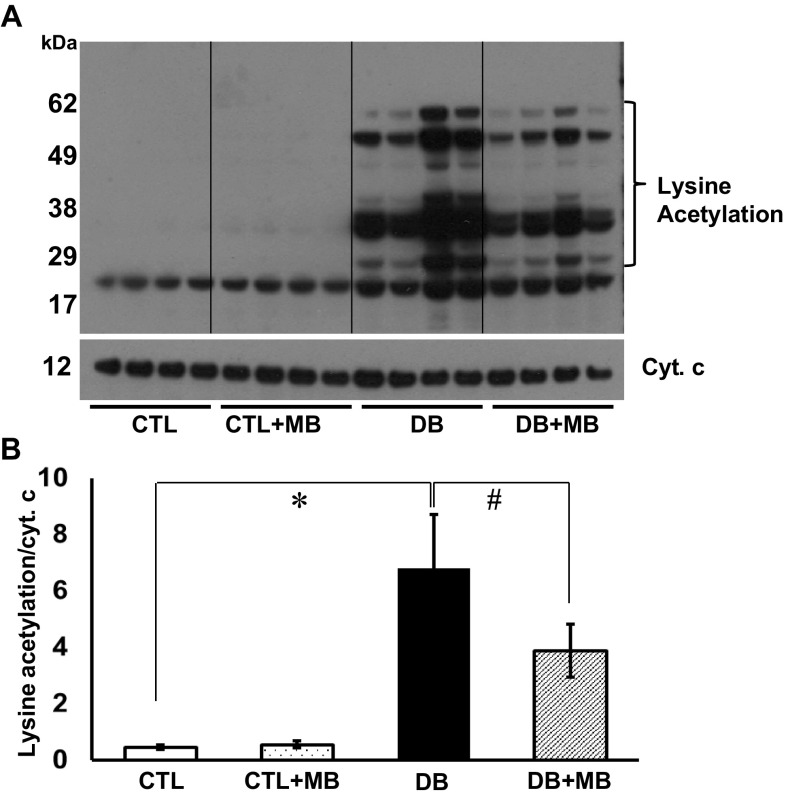
Methylene blue decreases protein lysine acetylation of cardiac mitochondria in diabetes. **a** Lysine acetylation modifications of protein extracts from isolated cardiac mitochondria. **b** Densitometric analysis was performed on the indicated portion of the membrane, and is shown as lysine acetylation reported to cytochrome c as the loading control. *p < 0.05 control (CTL, *N* = 4) versus diabetic (DB, *N* = 4), and ^#^ *p* < 0.05 diabetic (DB, *N* = 4) versus diabetic + methylene blue (DB+MB, *N* = 4). Mean ± SEM

**Fig. 2:**
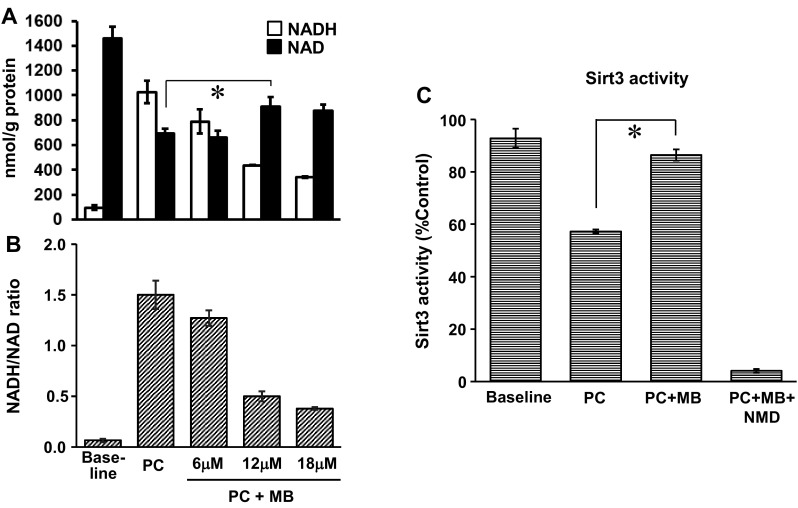
Methylene blue decreases NADH/NAD ratio and increases sirt3 activity in cardiac mitochondria. NADH and NAD^+^ amounts (**a**) and NADH/NAD^+^ ratio (**b**) in isolated naïve cardiac mitochondria oxidizing 0.04 mM palmitoylcarnitine (PC)+malate for 5 min. Methylene blue (MB) was added at the indicated concentrations (6, 12, and 18 μM). **c** Sirtuin 3 activity measured in cardiac mitochondria oxidizing 0.04 mM PC+malate. MB was added at 12 μM concentration. Nicotinamide (NMD) was added at 1 mM concentration. Data are the results of 3–5 independent experiments. *p < 0.05. Mean ± SEM

We previously reported an increase in lysine acetylation in cardiac mitochondria from DB rats despite unchanged expression of sirt3, and identified FA oxidation enzymes as major targets of this post-translational modification [13]. The present study confirms that finding, and further demonstrates that MB treatment decreases protein lysine acetylation in cardiac mitochondria (Fig. 1). Lysine acetylation is regulated by acetyl-CoA levels whereas deacetylation is regulated by NAD^+^-dependent deacetylases (sirtuins). We hypothesized that MB decreases lysine acetylation by changing the mitochondrial redox balance in favor of NAD^+^. NAD^+^ and NADH were quantitated and the NADH/NAD^+^ ratio was calculated in intact cardiac mitochondria isolated from naïve normal rats oxidizing palmitoylcarnitine (PC) as an energetic substrate (Fig. 2a, b). Mitochondrial redox status changed in favor of NADH upon oxidation of the energetic substrate. However, the addition of MB (6–18 μM) during substrate oxidation causes a dose-dependent decrease in NADH and the NADH/NAD^+^ ratio. Changes in the absolute amount of NAD^+^ were less responsive than NADH with MB addition; nevertheless, 12 μM MB led to a significant increase in [NAD^+^] confirming that MB shifts the mitochondrial redox status. To investigate the effect of this redox change on sirt3, its deacetylating activity was observed to be significantly increased in energized cardiac mitochondria with MB (12 μM) added (Fig. 2c). Nicotinamide (NMD), a sirt3 inhibitor, completely abolished the impact of MB on sirt3 activity attesting to the specificity of this result.

**Fig. 3:**
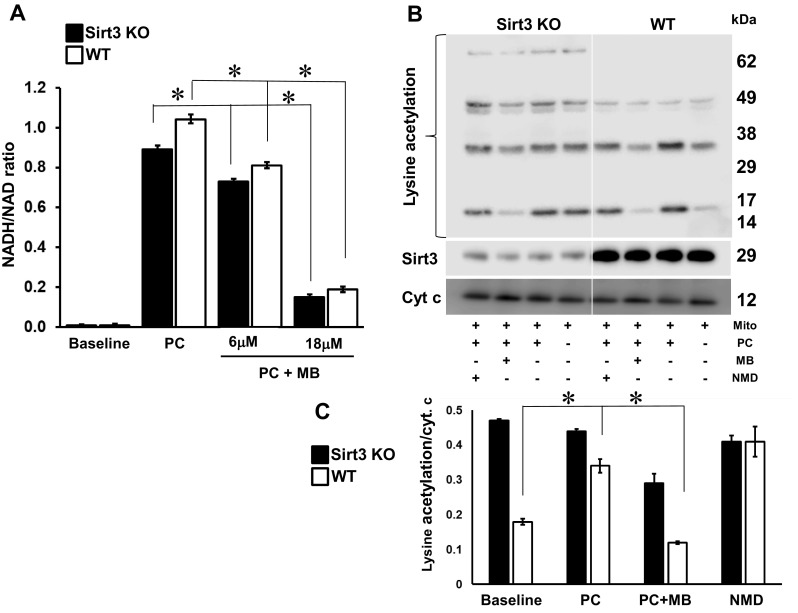
The lysine deacetylating effect of methylene blue is partially mediated by sirt3. **a** NADH/NAD^+^ ratio in cardiac mitochondria isolated from sirt3^−/−^ (sirt3 KO) and sirt3^+/+^ (wild-type, WT) mice. **b** Lysine acetylation modifications of cardiac mitochondrial proteins in sirt3^−/−^ (sirt3 KO) and sirt3^+/+^ (wild-type, WT) mice. **c** Densitometric analysis was performed on the indicated portion of the membrane, and is shown as lysine acetylation reported to cytochrome c as the loading control. Freshly isolated intact mitochondria (0.5 mg Mito) were incubated in either respiratory buffer alone (Baseline) or supplemented with palmitoylcarnitine (PC, 0.04 mM) + malate, PC+MB (12 μM), and PC+NMD (1 mM). At 5 min, mitochondrial proteins were extracted with lysis buffer, separated by PAGE (10 μg/lane) and probed with lysine acetylation, sirt3, and cytochrome c antibody. Data are the results of 1–3 independent experiments, *N* = 3 for either sirt3^−/−^ (sirt3 KO) and sirt3^+/+^ (wild-type, WT) mice. *p < 0.05. Mean ± SEM

To further investigate the dependency of MB’s deacetylating effect on sirt3 activity, cardiac mitochondria isolated from sirt3 KO mice were assessed in respiration studies, and changes in the redox status and protein acetylation were assessed (Fig. 3). Overall, energized mitochondria from sirt3 KO mice developed slightly lower NADH/NAD ratios (Fig. 3a). The addition of MB to PC-oxidizing mitochondria resulted in a dose-dependent decrease of NADH/NAD^+^ ratios in both WT and sirt3 KO cardiac mitochondria (Fig. 3a) that was primarily due to a decrease in NADH (Figure S2). Lysine acetylation of mitochondrial proteins was increased in sirt3 KO cardiac mitochondria compared to WT except under conditions where NMD was added to inhibit sirt3 (Fig. 3b, c). Furthermore, lysine acetylation was much less sensitive to the addition of MB in sirt KO cardiac mitochondria compared to WT (Fig. 3c). We show that whereas the MB-induced changes in the NADH/NAD ratio are largely independent on sirt3, the deacetylating effect of MB is dependent on the mitochondrial deacetylase sirt3. Taken together, these data support the link between the shift in mitochondrial redox status caused by MB, the induction of sirt3 activity, and the decrease in lysine acetylation of mitochondrial proteins.

### MB alters the global profile of mitochondrial protein acetylation with FA oxidation enzymes being the major targets

To evaluate individual proteins with lysine acetylation sites altered by MB treatment in diabetes, a proteomic analysis was performed on cardiac mitochondria from DB and DB+MB rats. We identified 1129 peptides representing 117 proteins present in both DB and DB+MB mitochondria, and detected 83 lysine acetylation sites on 34 proteins (Additional file: Table S2 presents the full proteomic survey). Acetylation of nearly all enzymes involved in mitochondrial FA transport and oxidation were affected by MB; the extent of lysine acetylation in the DB+MB group was lower compared to the same sites in the DB samples (Table 2). Relative differences in protein expression were much smaller than the changes in acetylated peptide amounts indicating that the decrease in acetylation is not due to altered protein abundance (Table S2). Interestingly, six lysine residues of the mitochondrial trifunctional protein subunit α, a protein complex which possesses FA oxidation activities, show decreased acetylation in the DB+MB group. Alignment of homologs across several other mammalian species demonstrates that four lysine residues (K60, 519, 569, and 728) are highly conserved (Fig. 6b).

**Table 2:** Cardiac mitochondrial proteins that are hypoacetylated at lysine residues in diabetic+methylene blue (DB+MB) versus diabetic (DB) group

Protein name and abbreviation/peptide sequence	Swiss-Prot accn#	Lysine position	Acetylation ratio
Electron transport chain			
ATP synthase subunit alpha (ATP5a1)	P15999		
K.GPVGS**K**IR.R		167	**0.50**
R.FNDGTDE**K**KK.L		239	**1.02**
R.SDG**K**ISEQSDAK.L		531	**0.24**
ATP synthase subunit beta (ATP5b)	P10719		
K.VLDSGAPI**K**IPVGPETLGR.I		133	**0.35**
ATP synthase subunit b (AT5f1)	P19511		
K.AQQALVQ**K**R.H		162	**0.38**
R.E**K**AQQALVQK.R		154	**0.28**
ATP synthase subunit gamma (ATPg)	P35435		
K.I**K**SILYR.T		113	**0.34**
K.SI**K**NIQK.I		14	**0.40**
R.THSDQFLVSF**K**DVGR.K		129	**0.40**
ATP synthase subunit d (ATP5h)	P31399		
K.YNAL**K**IPVPEDK.Y		78	**0.16**
R.ANVD**K**PGLVDDFK.N		63	**0.34**
R.ANVDKPGLVDDF**K**NK.Y		71	**0.26**
ATP synthase subunit O (ATPo)	Q06647		
K.RLDQVE**K**ELLR.V		60	**0.33**
R.LDQVE**K**ELLR.V		60	**0.35**
ADP–ATP translocase 1 (ANT1)	Q05962		
K.DFLAGGIAAAVS**K**TAVAPIER.V		23	**0.37**
K.IF**K**SDGLK.G		166	**0.22**
R.YFPTQALNFAF**K**DK.Y		92	**0.79**
ADP–ATP translocase 2 (ANT2)	Q09073		
R.YFPTQALNFAF**K**DK.Y		92	**0.79**
Cytochrome b-c1 complex subunit 1 QCR2c1	Q68FY0		
K.ALS**K**DLPK.V		38	**1.51**
Cytochrome b-c1 complex subunit 2 (QCR2c2)	P32551		
K.GASSF**K**ITR.G		97	**0.23**
R.GGLGLAGA**K**AK.Y		149	**0.34**
R.LASTLTT**K**GASSFK.I		91	**0.20**
R.SQL**K**IDK.A		159	**0.39**
Cytochrome c oxidase subunit 4 isoform 1 (COX41)	P10888		
K.LLSASQ**K**ALK.E		60	**0.25**
Glucose oxidation			
Dihydrolipoyl dehydrogenase (DLD)	Q6P6R2		
K.ALTGGIAHLF**K**QNK.V		143	**0.21**
K.ILGH**K**STDR.I		445	**0.23**
K.SEEQL**K**EEGVEFK.V		409	**0.15**
R.ILQ**K**QGFK.F		267	**0.20**
R.RPFTQNLGLEELGIELDP**K**GR.I		334	**0.29**
R.SAV**K**ALTGGIAHLFK.Q		132	**0.06**
Dihydrolipoyllysine-residue succinyltransferase component of 2-oxoglutarate dehydrogenase complex (ODO2)	Q01205		
R.DAFI**K**KN KDAFL**K**KH		273	**0.23**
R.H**K**DAFLK.K		268	**0.30**
R.H**K**DAFL**K**K.H		268, 273	**0.05**
FA oxidation			
2,4-dienoyl-CoA reductase (DECR)	Q64591		
K.QLI**K**AQK.G		185	**0.20**
Acetyl-CoA acetyltransferase (ACAT1)	P17764		
K.VL**K**YAGLK.K		335	**0.21**
K.YAGL**K**K.E		340	**0.24**
R.GATPYGGV**K**LEDLIVK.D		171	**0.16**
Acyl-coenzyme A thioesterase 1 (ACOT1)	O88267		
R.DE**K**GALFR.A		42	**0.20**
K.VPAS**K**AFTGFIVEADTPGIHIGK.K		217	**0.87**
Acyl-coenzyme A thioesterase 2 (ACOT2)	O55171		
R.DE**K**GALFR.A		83	**0.20**
R.DVQ**K**PYVVELEVLDGHEPDGGQR.L		141	**0.34**
Carnitine O-acetyltransferase (CRAT)	Q704S8		
R.VPGL**K**QDSVVNFLK.S		186	**0.12**
Enoyl-CoA delta isomerase 1 (ECI1)	P23965		
K.LEND**K**SIR.G		76	**0.23**
Hydroxyacyl-coenzyme A dehydrogenase (HADH)	Q9WVK7		
K.TLSSLSTSTDAASVVHSTDLVVEAIVENL**K**LK.N		125	**0.91**
Long-chain specific acyl-CoA dehydrogenase (LCAD)	P15650		
K.AFG**K**TVAHIQTVQHK.L		322	**0.33**
Medium-chain specific acyl-CoA dehydrogenase (MCAD)	P08503		
K.VPAS**K**AFTGFIVEADTPGIHIGK.K		217	**0.87**
Short-chain specific acyl-CoA dehydrogenase (SCAD)	P15651		
R.HAFGAPLT**K**LQNIQFK.L		306	**0.31**
Trifunctional enzyme subunit alpha (HADHa)	Q64428		
K.DTTASAVAVGL**K**QGK.V		531	**0.21**
K.TS**K**DTTASAVAVGLK.Q		519	**0.14**
R.DSIFSNLIGQLDY**K**GFEK.A		436	**0.47**
R.FVDLYGAQ**K**VVDR.L		728	**0.29**
R.ILQEGVDP**K**K.L		569	**0.15**
R.INSPNS**K**VNTLNK.E		60	**0.34**
Trifunctional enzyme subunit beta (HADHb)	Q60587		
R.TNIP**K**DVVDYIIFGTVIQEVK.T		96	**0.04**
TCA cycle			
Aconitate hydratase (ACO2)	Q9ER34		
K.FNPETDFLTG**K**DGK.K		517	**0.22**
K.IHPVD**K**LTIQGLK.D		723	**0.22**
R.AIIT**K**SFAR.I		689	**0.36**
R.YDLLE**K**NINIVR.K		50	**0.38**
Aspartate aminotransferase (GOT2)	P00507		
K.AEAQIAG**K**NLDK.E		90	**0.21**
R.FF**K**FSR.D		150	**0.30**
R.**K**AEAQIAGK.N		82	**0.22**
R.KAEAQIAG**K**NLDK.E		90	**0.14**
R.TQLVSNL**K**K.E		363	**0.23**
Fumarate hydratase	P14408		
K.**K**PVHPNDHVNK.S		170	**0.28**
Isocitrate dehydrogenase [NADP] (IDH2)	P56574		
K.DIFQEIFD**K**HYK.T		272	**0.24**
K.HY**K**TDFDK.N		275	**0.23**
K.LILPHVDVQL**K**YFDLGLPNR.D		80	**0.33**
K.LVFTP**K**DGSGAK.E		199	**0.28**
K.TDFD**K**NK.I		280	**0.20**
R.DQTNDQVTIDSALATQ**K**YSVAVK.C		106	**0.48**
R.FKDIFQEIFD**K**HYK.T		269	**0.18**
R.G**K**LDGNQDLIR.F		384	**0.28**
R.HAHGDQY**K**ATDFVVDR.A		180	**0.26**
Malate dehydrogenase (MDH2)	P04636		
K.GLE**K**NLGIGK.I		301	**0.28**
K.**K**GEDFVK.N		329	**0.19**
K.KGLE**K**NLGIGK.I		301	**0.34**
K.**K**HGVYNPNK.I		157	**0.28**
K.NLGIG**K**ITPFEEK.M		307	**0.25**
R.ANTFVAEL**K**GLDPAR.V		185	**0.23**
Other			
ES1 protein homolog	P56571		
K.GVTEAHVDQ**K**NK.V		131	**0.33**
NEDD8-conjugating enzyme (UBE2f)	Q5U203		
R.VSVRD**K**LLV**K**.E		35, 39	**0.29**
Phenylalanine–tRNA ligase alpha subunit (FARSa)	Q505J8		
R.VD**K**SAADGPR.V		117	**0.88**
Stress-70 protein (HSPa9a)	P48721		
R.AQFEGIVTDLI**K**R.T		348	**0.64**
R.HIV**K**EFK.R		288	**0.20**

*Bold* and *underlined* acetylated lysine residue in analyzed peptide fragment

Lysine position given refers to full protein sequence

Acetylation ratio reported as DB−MB:DB

### MB decreases long-chain FA β-oxidation without correcting the diabetes-induced complex I- and II-supported mitochondrial oxidative phosphorylation

**Fig. 4:**
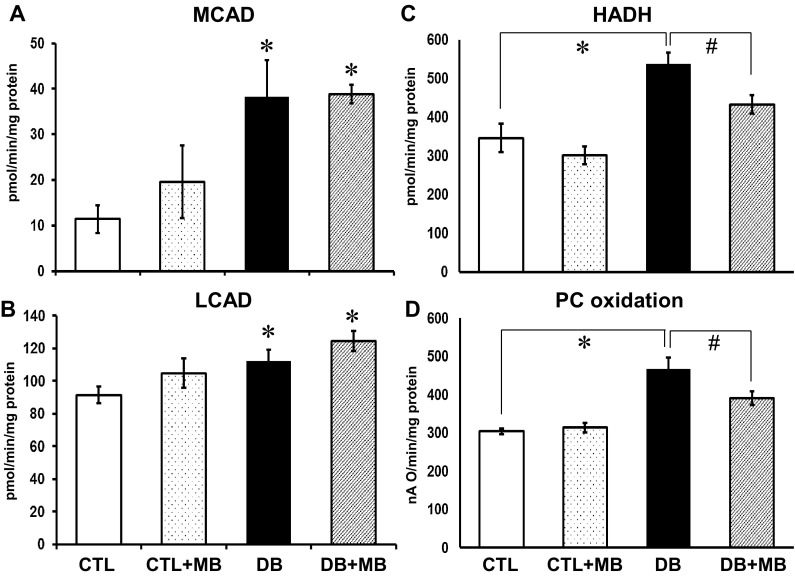
Methylene blue decreases mitochondrial fatty acid β-oxidation in diabetes. Specific activities of mitochondrial fatty acid β-oxidation enzymes were assessed spectrophotometrically in the presence of electron donors and acceptors in cardiac mitochondria. Medium-chain (**a**) and long-chain (**b**) acyl-CoA dehydrogenases (MCAD and LCAD) use the oxidation of specific substrates (octanoyl-CoA and palmitoyl-CoA) to reduce cytochrome c with phenazine ethosulfate as the intermediate electron acceptor from FADH2 bound to the enzyme. **c** Long-chain hydroxyacyl-CoA dehydrogenase (α-chain of the mitochondrial trifunctional protein) was measured as the backward conversion of acetoacetyl-CoA to hydroxyacetyl-CoA and NADH oxidation. **d** Mitochondrial fatty acid β-oxidation was measured as the maximal oxidation of PC+malate by intact cardiac mitochondria in the presence of 2 mM ADP. *p < 0.05 control (CTL, *N* = 4–7) versus diabetic (DB, *N* = 4–7), and ^#^ *p* < 0.05 DB (*N* = 4–7) versus diabetic+MB (DB+MB, *N* = 4–7). Mean ± SEM

In order to define whether MB alters FA oxidation in the DB heart, the specific activities of several enzymes were determined in cardiac mitochondria (Fig. 4). The activity of medium- and long-chain acyl-CoA dehydrogenase (MCAD and LCAD, respectively) increased with DB (Fig. 4a, b, respectively). HADH activity was also increased in DB mitochondria (Fig. 4c). Additionally, oxidation of PC (a long-chain acyl-CoA derivative) increased in the DB group (Fig. 4d). MB treatment did not alter MCAD and LCAD activities in the DB group, however, it significantly decreased HADH activity and PC oxidation (Fig. 4c, d). Our data on enzymes involved in mitochondrial FA oxidation suggest a preferential effect of MB on HDH leading to a decrease in its activity.

**Fig. 5:**
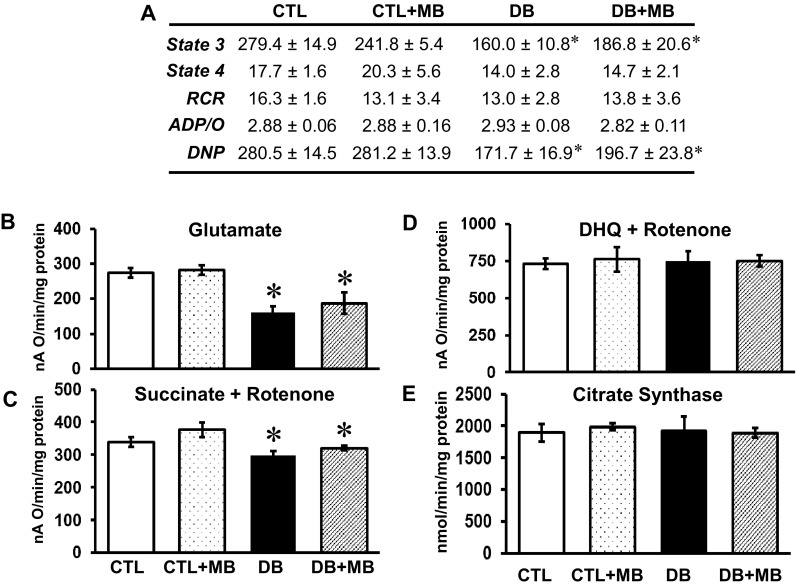
Methylene blue does not correct the diabetes-induced decrease in complex I- and II-supported oxidative phosphorylation in cardiac mitochondria. **a** Glutamate was used as substrate. State 3 respiratory rate (ADP-dependent) was induced by 0.2 mM ADP. State 4 respiratory rate (ADP-limited) was recorded after ADP consumption. RCR-Respiratory Control Ratio (State 3/State 4). ADP/O is the number of ADP molecules added for each oxygen atom consumed. Respiratory rates are expressed as nAO/min/mg mitochondrial protein. Maximal (2 mM) ADP-stimulated respiratory rates were measured with substrates that donate the reducing equivalents to complexes I (glutamate, **b**), II (Succinate+Rotenone, **c**), and III (DQ+R, Duroquinol+Rotenone, **d**). **e** Specific activity of citrate synthase. *p < 0.05. CTL = Control (*N* = 7), CTL+MB = Control+methylene blue (*N* = 7), DB = Diabetic (*N* = 7), DB+MB = Diabetic + methylene blue (*N* = 7). Mean ± SEM

To further test the effect of long-term MB treatment on cardiac mitochondrial function in DB rats, mitochondrial respiratory studies were performed on freshly isolated cardiac mitochondria. State 3 respiratory rate (ADP-dependent, 0.2 mM ADP) with glutamate was decreased in the DB group and was not significantly altered by MB treatment (Fig. 5a). State 4 respiratory rates (ADP-limited) were unchanged by DB as were respiratory control (RCR, state3/state4) and ADP/O ratios, indicating preserved respiratory coupling. Treatment with MB did not alter RCR or ADP/O. Depressed glutamate oxidation was insensitive to the dissipation of mitochondrial potential with the uncoupler dinitrophenol (DNP), excluding the phosphorylation system (phosphate transporter, ATP synthase, and adenine nucleotide translocator) as the defective component. Oxidative glutamate rates with high ADP (2 mM) were also decreased (Fig. 5b), congruent with the low ADP-induced state 3 respiration, indicating that the kinetic properties of the adenine nucleotide translocator are not responsible for the decreased oxidative phosphorylation in DB. These data confirm our previous observation that NADH oxidation by complex I, rather than NADH formation (upon glutamate oxidation) is affected in DB (Fig. 5b). State 3 respiratory rates with succinate were also lower in the DB group versus CTL, and were not improved by MB treatment (Fig. 5c). However, respiratory state 3 rates supported by duroquinol (DHQ, complex III electron donor) (Fig. 5d) and the cytochrome c electron donor, TMPD–ascorbate (not shown, and [13]), were not altered by DB, indicating functionally intact complexes III and IV. No differences were noted in the purity of mitochondrial fractions as indicated by citrate synthase activity (Fig. 5e). Our data confirm the complex I and II defects in the DB heart, and show that MB treatment does not ameliorate respiratory rate changes. Therefore, the observed MB-induced cardioprotection must occur through a mechanism not directly related to the intrinsic functional capacity of mitochondrial ETC.

## Discussion

Diabetic cardiomyopathy involves changes in the metabolic phenotype that are thought to precipitate cardiac dysfunction which eventually leads to heart failure. In the model used in this study, we observed key aspects of the human pathological condition associated with the development of diabetic cardiomyopathy. Heart rate was decreased in DB rats, consistent with the diabetic autonomic cardiac neuropathy with decreased adrenergic stimulation (50), and was not affected by MB. As we administered insulin to prevent hypercatabolism, modest systolic and diastolic dysfunction was evident in our study. A trend for improvement of all cardiac parameters was noted with MB treatment with significant improvement in cardiac volumes (systolic and diastolic) indicating improved whole organ function.

The normal heart is a high energy consumer, and generates ATP predominantly through mitochondrial oxidative phosphorylation, primarily through oxidation of FA [38]. Despite a heavy dependence on FA, energy substrate flexibility is critical in maintaining ATP levels to sustain heart function; overreliance on FA oxidation in the DB heart promotes a pathological metabolic inflexibility. Therapeutic strategies to fine-tune metabolism hold promise in treating diabetic cardiomyopathy but, pharmacological approaches to decrease circulating FA or partially inhibit cardiac FA uptake/β-oxidation have failed in clinical trials [39] implying a greater complexity to the underlying pathology. Reactive molecular species including acetyl-CoA and reducing equivalents (NADH) result from increased lipid oxidation, and are suggested to contribute to cardiac pathology [40].

Mitochondrial dysfunction, particularly manifested as a defect in complex I of the ETC [13], is believed to be a key mechanism in diabetic heart disease. However, complex I defect alone does not elicit an energetic deficit or cardiac dysfunction under resting, non-stressing conditions [41]. In contrast, the complex I defect in DB is not a limiting factor in the observed increase in FA oxidation in the diabetic heart and does lead to cardiac disease [13], indicating that the underlying molecular mechanism of DB cardiomyopathy extends beyond a decrease in ETC function and energy deficit. We previously demonstrated that the complex I defect in diabetes is associated with enhanced protein lysine acetylation [42]. While MB acts as an alternative electron carrier in complex I defective mitochondria and cells by bypassing complex I and provides neuroprotection in a murine model of complex I defect [19], evidence that it provides a similar electron route and rescues the complex I defect in the diabetic heart mitochondria [42] is missing. When isolated from the MB-treated diabetic hearts, cardiac mitochondria did not exhibit an improvement in complex I-dependent oxidative phosphorylation suggesting that the type of diabetic-induced complex I defect cannot be corrected by MB. In contrast, we observed the MB’s deacetylating effect in cardiac mitochondria after isolation, and reproduced it in in vitro experiments. These data indicate that lysine acetylation is a stable change, and that MB exerts a deacetylating effect disregarding the type of complex I defect.

Complex I not only participates in electron transfer for oxidative phosphorylation, but controls mitochondrial redox status by oxidizing NADH [41]. Indeed, mitochondria in the resting state favor NAD^+^, but when oxidizing an energetic substrate such as the long-chain FA derivative PC, NADH is relatively high compared to ETC flux and ATP synthesis. This redox shift towards NADH diminishes in the presence of an alternative electron carrier for complex I, MB, leading to increased [NAD^+^]. As NAD^+^ is the substrate and a potent activator of sirt3, the NADH to NAD^+^ shift with MB should directly increase sirt3 deacetylation activity. Indeed, our data from substrate-oxidizing mitochondria demonstrate increased sirt3 activity with the addition of MB. Sirtuin 3 (sirt3), the master regulator of mitochondrial deacetylation, has been classically recognized as a NAD^+^-reliant deacetylase and negative regulator of cardiac hypertrophy [43, 44]. Recent studies using in vitro approaches have suggested that NADH also inhibits sirt3 at concentrations that are non-physiologic [41, 45]. This has led to the notion that NADH does not likely regulate sirt3 activity in vivo [45]. However, early work on sirtuins hinted at a role for inhibition by [NADH] in yeast where NAD^+^/NADH ratios changed without altered NAD^+^; this was accompanied by sirtuin activation (with supporting in vitro data in the micromolar range) [46]. More recently, intact mitochondria from mice bearing a genetic defect in complex I, the major NADH oxidation site, do not alter NAD^+^ concentrations but rather show a significant increase in NADH compared to WT mice and a dramatic increase in lysine acetylation of mitochondrial proteins. Based on these in vivo findings and our previous work showing that decreased NADH consumption at complex I leads to elevated mitochondrial lysine acetylation in the DB heart [42], we assert that NADH also affects sirt3 activity. Our present data concurs as NADH was more responsive than NAD^+^ to MB at µM concentrations in respiring mitochondria (Fig. 2a). NAD^+^ also modestly but significantly increased at higher MB concentrations. MB also increased sirt3 activity (Fig. 2c). The link between NADH and sirt3 activity is further evidenced in mitochondria from WT and sirt3 KO mice where an MB-dependent decrease in the NADH/NAD^+^ ratio (Fig. 3a) was due to decreased NADH (Supplementary Fig. 2) and was accompanied by a suppression of acetylation only in WT; the absence of sirt3 abolished this effect (Fig. 3b, c). Though other factors that influence sirtuin activity warrant further investigation, our data firmly implicate a role for increased sirt3 activity as part of mechanism underlying MB-induced cardioprotection of the DB heart.

The data presented here with cardiac mitochondria from sirt3 KO mice demonstrate that mitochondrial redox state during substrate oxidation is modestly affected by the absence of sirt3, as previously reported [43]. Mitochondrial protein acetylation is increased in the absence of sirt3 and less sensitive to MB addition when compared to the WT mice. This effect is not evenly distributed as only the lysine acetylation of some proteins is sensitive to the addition of MB, an observation also confirmed by our proteomic approach. Our data imply that sirt3 activation is central to the lysine deacetylating effect of MB on some specific proteins, further linking mitochondrial redox status to the activation of sirt3. The absence of sirt3 does not fully abolish the effect of MB on acetylation suggesting that, although critical to mitochondrial protein deacetylation, sirt3 is not the sole deacetylase at least for some specific proteins. For example, lysine acetylation of the α subunit of trifunctional protein induced by sirt4, also a mitochondrial deacetylase, increases the protein stability by inhibiting ubiquitination, and promotes FA oxidation [47]. In contrast to this finding, sirt4 deacetylates and inhibits malonyl-CoA decarboxylase, an enzyme that converts malonyl-CoA, an inhibitor of mitochondrial FA oxidation, to acetyl-CoA, the carbon skeleton for lipogenesis; in this setting sirt4 limits mitochondrial FA oxidation [48]. These data emphasize the versatility of mitochondrial sirtuins to regulate FA metabolism. Also, MB-induced changes may be related to sirt3-mediated increases in antioxidant defense [44] which deserves further investigation. In systemic administration, MB is reported to be an inhibitor of guanylate cyclase [49] and reverses the nitric oxide-induced hypotension and vasoplegic syndrome [50], and acts as a redox compound in other tissues. As MB reportedly inhibits monoamine oxidase A [51], a source of oxidative stress in the diabetic heart, we cannot exclude the possibility that its antioxidant role may have also contributed to the improvement of cardiac function. Regardless, in our whole animal studies in a diabetic rat model, the increase in protein acetylation observed in DB was diminished by treatment with MB and cardiac function was improved; this underscores the physiological relevance of the biochemical effect of mitochondrial redox state on protein acetylation in the heart.

The functional consequences of lysine acetylation in the context of our biochemical data and the impact on FA oxidation is of interest in defining how DB and MB affect cardiac metabolism. First, we assessed mitochondrial FA oxidation through specific activities of several reactions involved in FA oxidation. As indicated by PC oxidation, overall FA oxidation was enhanced in DB hearts and MB treatment decreased oxidation, which is consistent with our hypothesis that MB should reduce FA metabolism, along with protein acetylation. The activities of MCAD and LCAD, enzymes catalyzing the initial steps of FA metabolism, were increased by DB consistent with increased FA oxidation. However, MB treatment did not affect MCAD and LCAD activities despite an anticipated decrease to accompany our observation of reduced protein acetylation. The activity of HADH, an enzyme catalyzing a downstream step of FA oxidation was also increased in the hearts of DB rats. In contrast to MCAD and LCAD, its activity was decreased with MB treatment. Together, the data suggest specific targeting of mitochondrial FA pathway proteins by MB-induced lysine deacetylation.

To gain more insight on MB-induced changes in lysine acetylation of mitochondrial proteins, a non-targeted approach was employed to generate a proteomic profile. MB decreases protein acetylation in a broad range of mitochondrial metabolic pathways including FA transport and oxidation, amino acid metabolism, tricarboxylic acid cycle, ETC, mitochondrial membrane transport, and regulatory proteins. However, the deacetylating effect is not evenly distributed (fold changes between 0.06 and 0.91). Processes related to FA-derived acetyl-CoA were heavily affected as numerous FA β-oxidation pathway enzymes had at least one lysine target. This included acetylation of three lysine residues in the short-, medium-, and long-chain acyl-CoA dehydrogenases that decreased with MB treatment. Although substrate length specificity varies, the catalytic mechanism of MCAD and LCAD is similar; all enzymes transfer protons from acyl-CoA to FAD with the reducing equivalents being transferred to the electron transfer flavoprotein with final disposition into the ETC via coenzyme Q. The activities of these enzymes in DB hearts were increased; however, MB did not alter acyl-CoA dehydrogenase activities despite decreasing lysine acetylation. It was previously reported that LCAD lysine residues 318 and 322 are sirt3 targets, and dual acetylation decreases activity [12]. We suggest that there may exist a synergistic effect of acetylating both lysine residues not present in our model, as increased acetylation occurred solely on lysine 322 in the DB group associated with increased enzyme activity.

**Fig. 6:**
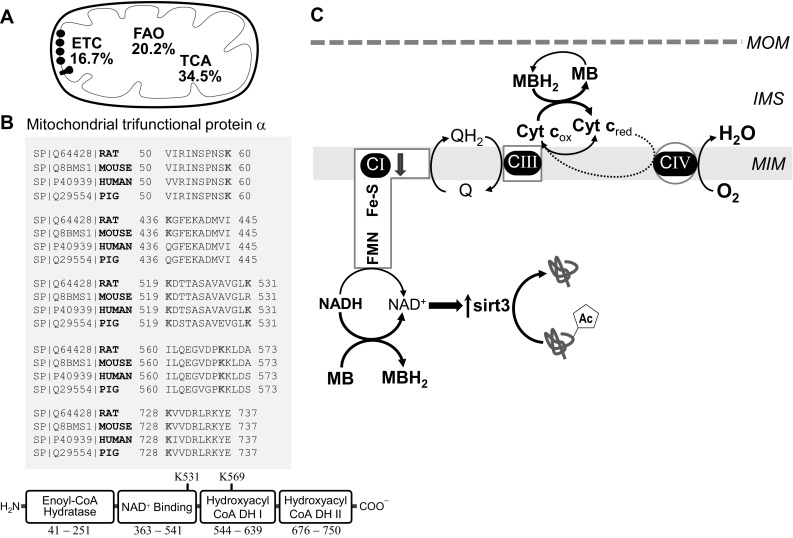
Methylene blue decreases mitochondrial lysine acetylation and relieves the metabolic inflexibility of the diabetic heart by facilitating NADH oxidation. **a** Target classification of MB-mediated decreases of protein lysine acetylation in diabetes. MB decreases lysine acetylation of proteins across several mitochondrial metabolic pathways including fatty acid transport and oxidation (FAO), tricarboxylic acid cycle (TCA), electron transport chain (ETC), mitochondrial membrane transport and regulatory proteins, and amino acid metabolism. **b** Multiple sequence alignment of the mitochondrial trifunctional protein α subunit. The mouse, human, and pig proteins were aligned with the rat protein. The alignment was created with CLUSTAL program available at http://www.uniprot.org/align. Domain was built based on information found at http://www.ncbi.nlm.nih.gov/Structure/cdd/wrpsb.cgi. **c** Proposed lysine deacetylating effect of MB in the diabetic heart. The diabetes-induced mitochondrial complex I defect limits the oxidation of NADH. MB facilitates NADH oxidation, thus increasing NAD^+^ and the activity of the NAD^+^-dependent deacetylase sirt3, and decreasing lysine acetylation of FA oxidation enzymes and metabolic inflexibility. *FMN* Flavin mononucleotide; Fe–S + Iron sulfur centers; * C I, III, and IV  *Complexes I, III, and IV; *Cyr c* Cytochrome c; *MOM* Mitochondrial outer membrane; *IMS* Intermembrane space; *MIM* Mitochondria inner membrane

A major target of decreased acetylation with MB treatment was the mitochondrial trifunctional protein complex (Fig. 6b), a large hetero-multimeric enzyme catalyzing the three final steps of long-chain FA β-oxidation. It comprised four α-subunits with long-chain enoyl-CoA hydratase and 3-hydroxyacyl-CoA dehydrogenase (HADH) activities, and four β-subunits harboring the long-chain 3-ketoacyl-CoA thiolase activity. The α subunit is reported to be heavily lysine acetylated normally [52] and in diabetes [13]. We found six lysines that exhibit decreased acetylation with MB treatment in DB suggesting finely tuned regulation through multiple acetylation sites; four of these lysines were identified by Rardin et al. as sirt3 targets [52]. However, lysines 531 and 569, located within the 3-hydroxyacyl-CoA dehydrogenase, are novel to this study. Lysines 60, 519, 569, and 728 are conserved among mammalian species, supporting a role in regulating function, though the functional consequences are a matter of debate. Hyper-acetylation inhibits the enzyme in mice lacking cyclophilin D [53], whereas it activates the enzyme in mice with high fat diet (HFD)-induced obesity [11]. The sites of acetylation have not been provided by these studies. Our study suggests that with increased FA availability, enhanced acetylation at lysines 531 and 569 is associated with enzyme activation in the DB heart. Our results also indicate that decreased lysine acetylation of FA oxidation enzymes with MB treatment positively correlates with a decrease in the overall FA β-oxidation pathway, suggesting a protective effect of this compound on the excessive reliance of the heart on FA oxidation in diabetes. Future studies will need to validate and elucidate how site-specific lysine acetylation modulates the enzyme function.

Currently there is no consensus regarding the effect of lysine acetylation on mitochondrial FA oxidation in the diabetic heart. Using approaches that overexpress or knockdown sirt3, FA oxidation enzymes are reported to be deacetylated and activated by sirt3 in the liver [12, 54]. However, cultured HEK293 cells exposed to histone deacetylase inhibitors exhibit an increased lysine acetylation and activation of enoyl-coenzyme A hydratase/3-hydroxyacyl-coenzyme A dehydrogenase in mitochondrial FA oxidation [55]. In addition, in a rodent model of T1D we found an increased lysine acetylation of FA oxidation enzymes in the heart in the presence of unchanged sirt3 amount and associated with increased mitochondrial FA oxidation supported by an increased activity of FA oxidation enzymes [42]. Similarly, HFD regimen led to increased lysine acetylation of FA oxidation enzymes and mitochondrial FA oxidation in the heart [11]. Therefore, mitochondrial FA oxidation enzymes are hyperacetylated and activated in the context of increased FA availability to the heart regardless of sirt3 amount suggesting that additional levels of regulation may operate during these conditions. Mechanisms that control this context-specific response have just begun to be investigated. One operative mechanism is provided by heat shock protein 10 (a sirt3 substrate) that, when acetylated, induces an optimal folding of the FA oxidation protein enzymes enhancing their activity and overall mitochondrial FA oxidation during HFD [56], a condition that is also associated with a decrease in the NAD^+^/NADH redox ratio and sirt3 activity.

In addition, the role of increased acetyl-CoA derived from the oxidation of excessive fuel substrates in diabetes (glucose, FA) has begun to be appreciated. The importance of acetyl-CoA is supported by experiments in mice bearing a deficiency of carnitine acetyltransferase that transports the excessive acetyl-CoA from mitochondria to cytosol; these mice exhibit increased mitochondrial lysine acetylation induced by excessive mitochondrial acetyl-CoA [57]. Acetyl-CoA is the substrate and acetyl group donor for acetyltransferases to acetylate lysine groups on proteins. The mitochondrial acetyltransferase, GCN5-1, counteracts the sirt3 deacetylating effect on mitochondrial proteins [58]. With low binding affinity for acetyl-CoA, all acetyltransferases are reliant on acetyl-CoA availability [59] and may be inhibited by CoA suggesting that the acetyl-CoA/CoA is equally important for lysine acetylation [60]. In addition, excessive acetyl-CoA can promote non-enzymatic lysine acetylation [61, 62]. Compounds resulting from oxidation of the large availability of fuel substrates, including acylcarnitines and acetyl-CoA, accumulates upon HFD [63]. Therefore, non-enzymatic lysine acetylation is operative during an excess energetic supply to the heart leading to acetylation of lysine sites other than those reported to be sirt3 targets. We propose that in the diabetic heart the non-enzymatic (and most likely non-specific) lysine acetylation activates mitochondrial FA oxidation and overrides the inhibitory effect of lysine acetylation at mitochondrial sirt3 lysine targets.

Collectively, our data support the concept that mitochondrial ETC dysfunction is an important arbitrator of diabetic cardiomyopathy, and leads to a shift in mitochondrial redox status that favors lysine acetylation of FA oxidation enzymes and contributes to the excessive reliance of FA oxidation in the diabetic heart. This work also highlights a novel therapeutic approach in diabetes; the use of mitochondrial alternative electron carriers to decrease lysine acetylation, and alleviate the metabolic rigidity and cardiac dysfunction of the diabetic heart. Figure 6c shows a proposed mechanism for the lysine deacetylating effect of MB. In the mitochondrial ETC, electrons are passed from NADH via several donors and acceptors to oxygen in order to form water. The energy obtained through this electron transfer is used by complexes I, III, and IV to pump protons into the intermembrane space creating an electrochemical gradient that is further used by ATP synthase to phosphorylate ADP to ATP. Diabetic-induced complex I defect causes a decrease in NADH oxidation. In the experimental models of rotenone-induced complex I defect MB accepts electrons from catalytic subunits of complex I and become reduced (MBH2) whereas cytochrome c reoxidizes MBH2 to MB [20]. Therefore, MB provides an alternative electron route within the diabetic complex I-deficient cardiac mitochondria and favors NADH oxidation, increased NAD^+^, and sirt3 activity.

MB was reported to accumulate with high affinity in tissues including liver and heart, increase the cellular NAD^+^/NADH ratio, and enhance sirt1 expression in the liver [29]. Our study adds to these findings and show that MB also accumulates within mitochondria, and establishes the causal link between the MB-induced decrease in the NADH/NAD^+^ ratio and activation of mitochondrial sirt3 with limitation of the specific activity of the hydroxyacyl-CoA dehydrogenase and FA β-oxidation, and functional improvement of the diabetic heart. We propose that MB-mediated protein deacetylation of FA metabolism enzymes is directly linked to improved cardiac function in DB. Whether this is the sole mechanism of cardioprotection by MB requires further investigation. Future work on site-directed mutagenesis of MB targeted-lysine residues will aid in establishing the relationship between this post-translational modification and the change in the enzyme-specific activity.

## Electronic supplementary material

Below is the link to the electronic supplementary material.

Supplementary material 1 (XLSX 15 KB)

Supplementary material 2 (XLSX 104 KB)

Supplementary material 3 (PPTX 378 KB)

Supplementary material 4 (PPTX 43 KB)

## Abbreviations

MB: Methylene blue
ETC: Electron transport chain
NADH: Nicotinamide dinucleotide reduced
NAD^+^: Nicotinamide dinucleotide oxidized
FA: Fatty acid
Sirt 3: Sirtuin 3
MCAD: Medium-chain acyl-CoA dehydrogenases
LCAD: Long-chain acyl-CoA dehydrogenases
HADH: Hydroxyacyl-coenzyme A dehydrogenase
PC: Palmitoylcarnitine
CTL: Control
CTL+MB: Control+Methylene blue
DB: Diabetic
DB+MB: Diabetic+Methylene blue
