# Supplementary material for: Methylene blue decreases mitochondrial lysine acetylation in the diabetic heart
Source: Mol Cell Biochem. 2017 Mar 16;432(1):7–24. doi: 10.1007/s11010-017-2993-1 (PMC5532421; doi:10.1007/s11010-017-2993-1)
Supplement: Supplementary file 3 — Supplementary material 3 (PPTX 378 KB) [file 11010_2017_2993_MOESM3_ESM.pptx]

## Slide 1
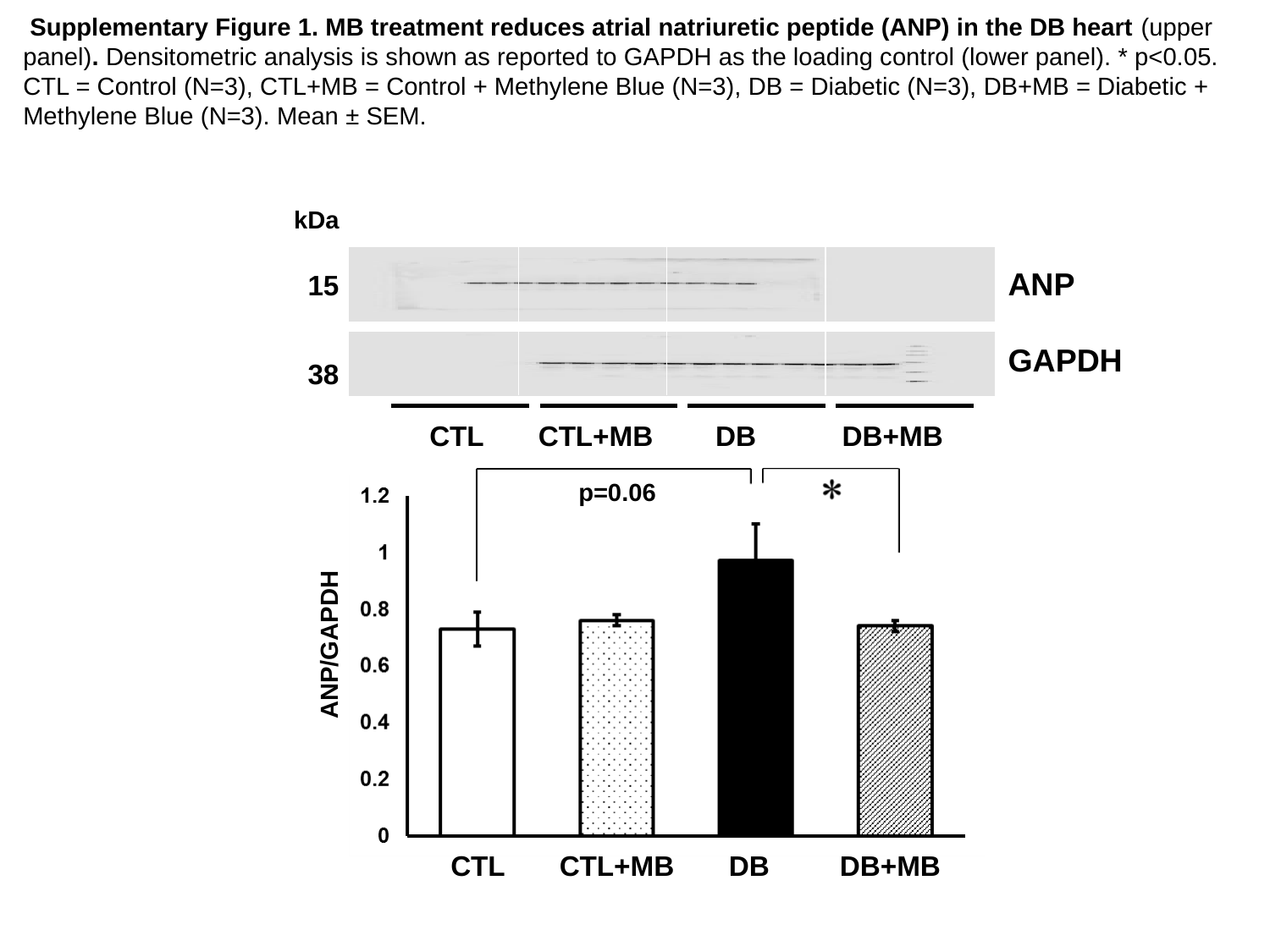

Supplementary Figure 1. MB treatment reduces atrial natriuretic peptide (ANP) in the DB heart (upper panel). Densitometric analysis is shown as reported to GAPDH as the loading control (lower panel). * p<0.05. CTL = Control (N=3), CTL+MB = Control + Methylene Blue (N=3), DB = Diabetic (N=3), DB+MB = Diabetic + Methylene Blue (N=3). Mean ± SEM.
kDa
15
38
ANP
GAPDH
 CTL CTL+MB DB DB+MB
p=0.06
ANP/GAPDH
 CTL CTL+MB DB DB+MB
