# Supplementary material for: Methylene blue decreases mitochondrial lysine acetylation in the diabetic heart
Source: Mol Cell Biochem. 2017 Mar 16;432(1):7–24. doi: 10.1007/s11010-017-2993-1 (PMC5532421; doi:10.1007/s11010-017-2993-1)
Supplement: Supplementary file 4 — Supplementary material 4 (PPTX 43 KB) [file 11010_2017_2993_MOESM4_ESM.pptx]

## Slide 1
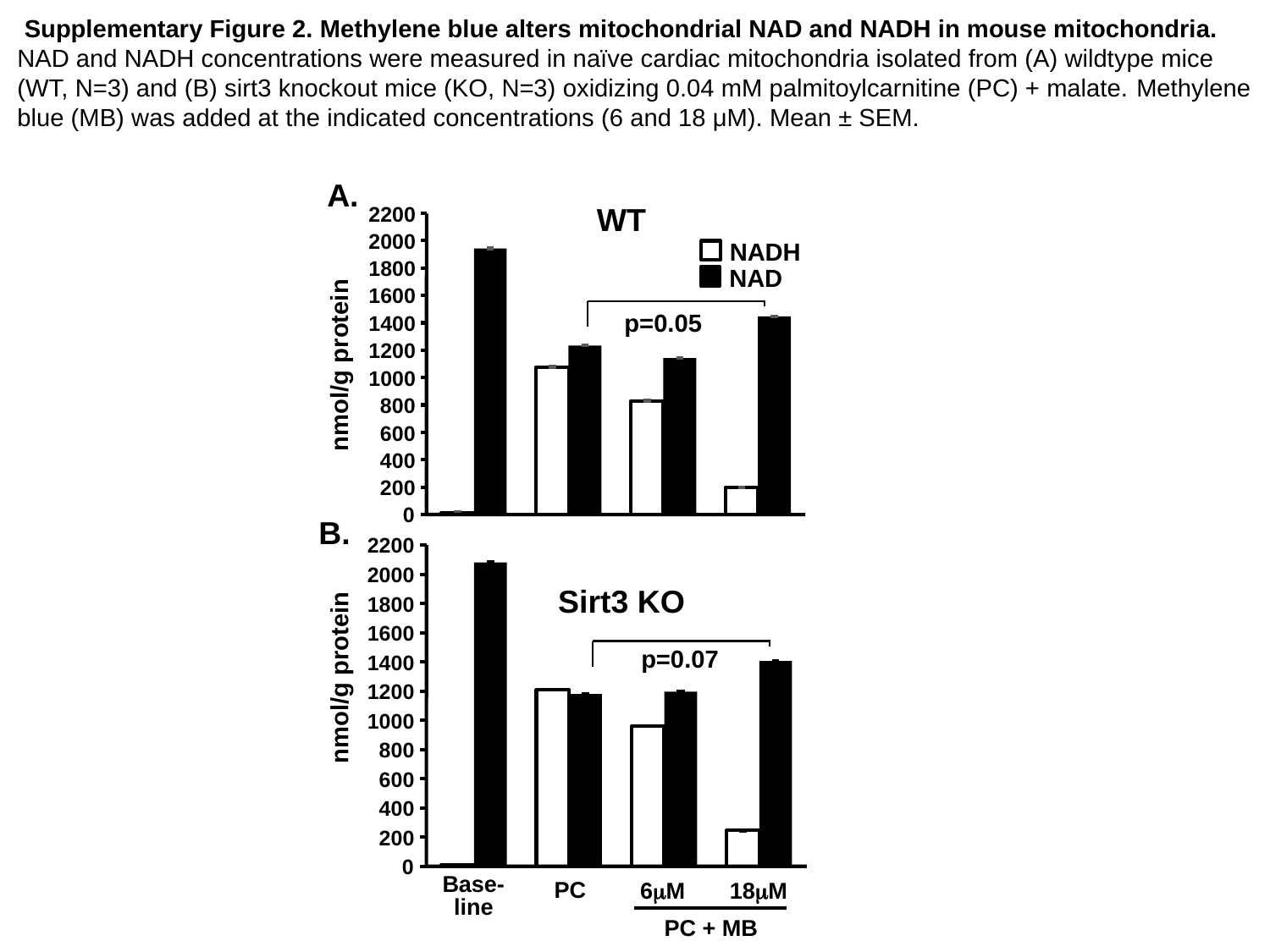

Supplementary Figure 2. Methylene blue alters mitochondrial NAD and NADH in mouse mitochondria. NAD and NADH concentrations were measured in naïve cardiac mitochondria isolated from (A) wildtype mice (WT, N=3) and (B) sirt3 knockout mice (KO, N=3) oxidizing 0.04 mM palmitoylcarnitine (PC) + malate. Methylene blue (MB) was added at the indicated concentrations (6 and 18 μM). Mean ± SEM.
A.
WT
2200
2000
1800
1600
1400
1200
1000
800
600
400
200
0
NADH
NAD
nmol/g protein
B.
2200
2000
1800
1600
1400
1200
1000
800
600
400
200
0
Sirt3 KO
nmol/g protein
Base-
line
PC
6μM
18μM
PC + MB
p=0.05
p=0.07
